# *Salmonella enterica* Pulsed-Field Gel Electrophoresis Clusters, Minnesota, USA, 2001–2007

**DOI:** 10.3201/eid1611.100368

**Published:** 2010-11

**Authors:** Joshua M. Rounds, Craig W. Hedberg, Stephanie Meyer, David J. Boxrud, Kirk E. Smith

**Affiliations:** Author affiliations: Minnesota Department of Health, St. Paul, Minnesota, USA (J.M. Rounds, S. Meyer, D.J. Boxrud, K.E. Smith);; University of Minnesota School of Public Health, Minneapolis, Minnesota, USA (C.W. Hedberg)

**Keywords:** Salmonella enterica, surveillance, serovars, outbreak, foodborne diseases, pulsed-field gel electrophoresis, clusters, bacteria, Minnesota, research

## Abstract

We determined characteristics of *Salmonella enterica* pulsed-field gel electrophoresis clusters that predict their being solved (i.e., that result in identification of a confirmed outbreak). Clusters were investigated by the Minnesota Department of Health by using a dynamic iterative model. During 2001–2007, a total of 43 (12.5%) of 344 clusters were solved. Clusters of >4 isolates were more likely to be solved than clusters of 2 isolates. Clusters in which the first 3 case isolates were received at the Minnesota Department of Health within 7 days were more likely to be solved than were clusters in which the first 3 case isolates were received over a period >14 days. If resources do not permit investigation of all *S. enterica* pulsed-field gel electrophoresis clusters, investigation of clusters of >4 cases and clusters in which the first 3 case isolates were received at a public health laboratory within 7 days may improve outbreak investigations.

## Abstract 2

This procedure can help identify outbreaks of salmonellosis.

Salmonellosis is a major foodborne illness that results in ≈1.4 million infections, 15,000 hospitalizations, and 400 deaths each year in the United States (*1**,**2*). *Salmonella* infections are primarily of foodborne origin but can also occur through contact with infected animals, humans, or their feces (*3*). The epidemiology of salmonellosis is complex largely because there are >2,500 distinct serotypes (serovars) with different reservoirs and diverse geographic incidences (*4*). Changes in food consumption, production, and distribution have led to an increasing frequency of multistate outbreaks associated with fresh produce and processed foods (*5*).

The development of molecular subtyping by pulsed-field gel electrophoresis (PFGE) has revolutionized *Salmonella* spp. surveillance. The National Molecular Subtyping Network for Foodborne Disease Surveillance (PulseNet) provides state and local public health department laboratories with standardized methods to subtype *Salmonella* serovars and normalize PFGE patterns against a global reference standard provided by the Centers for Disease Control and Prevention (CDC) (*6**,**7*). Molecular subtyping enhances case definition specificity, enabling outbreaks to be detected and controlled at an earlier stage, and enabling detection of geographically dispersed outbreaks (*8**–**10*).

Although the benefits of molecular subtyping, specifically by PFGE, in foodborne disease outbreak detection and investigation have been well established, there is no consensus about when a PFGE cluster warrants further investigation and almost no quantitative analysis about characteristics of PFGE clusters that indicate a common source will be identified (*11**–**15*). Cluster size and the number of days from receipt of the first cluster case isolate to the third case isolate received by the public health laboratory were predictors of a source of infection being identified for *Listeria monocytogenes* clusters in France (*16*). The objective of this study was to determine characteristics of *Salmonella* PFGE clusters that could serve as useful predictors for their being solved (i.e., result in identification of a confirmed outbreak). This information could help public health agencies with limited resources prioritize investigation of *Salmonella* PFGE clusters.

## Materials and Methods

*Salmonella* infections are reportable to the Minnesota Department of Health (MDH) by state law (*17*). Clinical laboratories are required to forward all *Salmonella* isolates to the MDH Public Health Laboratory (PHL). PFGE subtyping after digestion with *XbaI* is conducted on all isolates as soon as they are received according to PulseNet protocols (*18*). PFGE subtypes are uploaded into the national PulseNet database (*6*). All Minnesota residents with a culture-confirmed *Salmonella* infection are routinely interviewed as soon as possible by MDH staff with a standard questionnaire about symptom history, food consumption, and other potential exposures occurring in the 7 days before onset of illness. The questionnaire contains detailed food exposure questions, including open-ended food histories and objective yes/no questions about numerous specific food items, as well as brand names and purchase locations. Clusters are investigated by using an iterative model in which suspicious exposures identified during initial case-patient interviews are added to the standard interview for subsequent cases (*19**–**21*). Similarly, initial cluster case-patients may be reinterviewed to ensure uniform ascertainment of the suspicious exposures. This iterative approach is used to identify exposures for further evaluation with formal hypothesis testing, product sampling, or product tracing (*19*).

A cluster was defined as >2 cases of salmonellosis in different households with isolates of the same serovar and PFGE subtype and with specimen collection dates within 2 weeks (*22*). Thus, a single cluster would be ongoing as long as a new isolate was collected within 2 weeks after the most recent isolate in the cluster. A cluster was considered solved if the epidemiologic evaluation of that cluster resulted in the identification of a common source of infection for those cases and consequently the documentation of a confirmed outbreak. Therefore, the terms solved cluster and confirmed outbreak are equivalent and used interchangeably.

### Inclusion and Exclusion Criteria

Laboratory-confirmed cases of nontyphoidal *Salmonella enterica* infection among Minnesota residents with specimen collection dates from January 1, 2001, through December 31, 2007, for which isolates were received and subtyped by MDH PHL were included in the study. Isolates not received through routine surveillance (i.e., testing was requested or conducted by MDH as a part of an ongoing investigation) were excluded from the analysis.

Solved clusters were included if they were detected and identified solely on the basis of investigation of cases identified through submission of isolates to MDH for routine laboratory surveillance. Solved clusters for which a call to the MDH foodborne disease hotline (www.health.state.mn.us/divs/idepc/dtopics/foodborne/reporting.html) (e.g., from the public or a healthcare provider) directly contributed to the identification of an outbreak were excluded from analysis. Secondary clusters, defined as clusters in which the cases were part of a confirmed outbreak that had been previously identified, were also excluded from analysis. Clusters that were part of a probable outbreak (an epidemiologic evaluation suggested, but did not confirm, a common source of infection) were also excluded.

### Study Variables

Variables incorporated into the analysis were cluster year, cluster size, cluster case density, cluster serovar, cluster subtype, and cluster serovar diversity. Cluster size was defined as the number of cases in each cluster and was categorized into cluster sizes of 2, 3, 4, and >5. For clusters in which a common source was identified, only cases received before the cluster was solved were included. Cluster case density was defined as the number of days from receipt date of the first cluster isolate at MDH PHL to the receipt date of the third cluster isolate and was categorized into cluster case densities of 0, 1–7, 8–14, and >14 days (*16*).

Cluster serovar was coded as a categorical variable on the basis of serovar frequency. Serovars representing >20% of all isolates (Typhimurium and Enteritidis) were categorized as very common, those representing 3%–20% (Newport, Heidelberg, and Montevideo) as common, and those representing <3% (all other serovars) as uncommon. The relationship between common and uncommon PFGE subtypes and solving a cluster was examined for serovars Typhimurium and Enteritidis. For serovar Typhimurium, clusters with CDC PFGE subtype designations JPXX01.0003, JPXX01.0410, and JPXX01.0111 (each representing >8% of all Typhimurium isolates) were categorized as common, and all other subtypes were categorized as uncommon. For serovar Enteritidis, clusters with CDC PFGE subtype designations JEGX01.0004 and JEGX01.0030 (each representing >20% of all Enteritidis isolates) were categorized as common, and all other subtypes were categorized as uncommon.

Cluster serovar diversity was examined by categorizing the 17 most frequent serovars into highly clonal or low clonality serovars on the basis of the Simpson diversity index (*23*). Serovars with a Simpson index score <0.90 were considered highly clonal, and serovars with a Simpson index score >0.90 were considered to have low clonality. Cluster investigation thresholds were examined by comparing the percentage of outbreak clusters meeting a threshold, cluster investigation positive predictive value, and estimated interview burden in hours per year for various investigational thresholds. The time required to interview each patient with a *Salmonella* infection by using the MDH standard questionnaire was recorded for a 6-month period in 2008, and the median interview time was calculated.

### Statistical Analysis

A descriptive analysis was conducted to characterize the frequency of *Salmonella* serovars and subtypes. Mantel-Haenszel χ^2^ test for trend was used to characterize temporal trends in the number of *Salmonella* clusters that were solved. Two-sided Wilcoxon rank-sum tests were used to compare the median cluster size and cluster density of point source and non–point source outbreaks. Univariate analysis was performed to calculate odds ratios (ORs) and 95% confidence intervals (CIs) characterizing the crude associations between *Salmonella* cluster serovar, cluster PFGE subtype, cluster serovar diversity, cluster size, and cluster case density and a cluster being solved. Mantel-Haenszel χ^2^ tests for trend and interaction terms were used to investigate the linear nature of the relationship between cluster size, cluster case density, and the outcome. SAS software version 9.1 (SAS Institute, Cary, NC, USA) was used for descriptive and univariate analysis. An α value <0.05 was considered significant.

## Results

**Figure 1:**
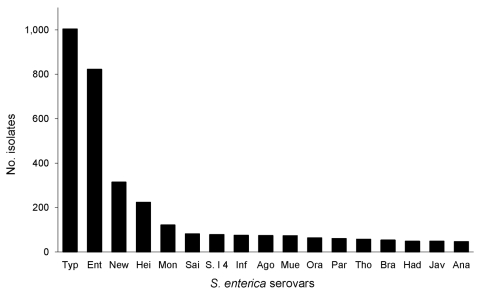
Frequency of the 17 most common *Salmonella enterica* serovars among clinical case isolates submitted to the Minnesota Department of Health, 2001–2007. Typ, Typhimurium; Ent, Enteritidis; New, Newport; Hei, Heidelberg; Mon, Montevideo; Sai, Saintpaul; S.I4, S.I 4,5,12:I:­–; Inf, Infantis, Ago, Agona; Mue, Muenchen; Ora, Oranienburo; Par, Paratyphi B var. L; Tho, Thompson; Bra, Braenderup; Had, Hadar; Jav, Javiana; Ana, Anatum.

During 2001–2007, a total of 4,154 nontyphoidal *Salmonella* isolates from Minnesota residents were received at MDH through routine surveillance; they represented 98% of reported *Salmonella* cases (n = 4,235, incidence 11.78 cases/100,000 person-years). PFGE subtyping was performed for 4,018 (97%) isolates, which were included in the study. Among these isolates, 194 *Salmonella* serovars were observed. The 6 most common *S*. *enterica* serovars were Typhimurium, 1,004 (25%); Enteritidis, 822 (20.5%); Newport, 314 (7.8%); Heidelberg, 223 (5.6%); Montevideo, 121 (3.0%); and Saintpaul, 81 (2.0%) (Figure 1).

The frequency of PFGE subtypes was examined in detail for serovars Typhimurium and Enteritidis. The 3 most common subtypes of serovar Typhimurium were JPXX01.0003, 107 (11%); JPXX01.0410, 87 (9%); and JPXX01.0111, 85 (8%). The 3 most common subtypes of serovar Enteritidis were JEGX01.0004, 309 (38%); JEGX01.0030, 181(22%); and JEGX01.0005, 106 (13%).

Serovar diversity was examined by comparing Simpson diversity indices for the 17 most frequent serovars (Table 1). Javiana, Newport, Agona, Infantis, and Typhimurium were low clonality serovars. Heidelberg, Hadar, Enteritidis, Thompson, and I 4,5,12:I:– were highly clonal serovars.

**Table 1:** *Salmonella enterica* serovar diversity identified by pulsed-field gel electrophoresis among case isolates submitted to the Minnesota Department of Health, 2001–2007*

Serovar	No. isolates	No. PFGE subtypes observed	Serovar isolates represented by most common subtype, %	Serovar isolates represented by 2 most common subtypes, %	Serovar isolates represented by 3 most common subtypes, %	Simpson index†
Heidelberg	223	46	57	62	66	0.67
Hadar	48	20	48	54	58	0.77
Enteritidis	822	80	38	60	73	0.79
Thompson	57	23	42	53	58	0.81
I 4,5,12:I:–	78	25	31	50	60	0.86
Braenderup	53	30	26	36	43	0.92
Oranienburg	63	26	21	32	41	0.93
Anatum	46	22	17	33	46	0.93
Paratyphi B var. L	60	35	22	37	43	0.93
Montevideo	121	59	22	30	36	0.94
Muenchen	73	50	21	25	27	0.96
Saintpaul	81	44	17	26	32	0.96
Typhimurium	1,004	285	11	20	28	0.96
Infantis	75	43	9	17	24	0.97
Agona	74	48	10	16	22	0.98
Newport	314	143	10	15	19	0.98
Javiana	48	41	6	11	15	0.99

*PFGE, pulsed-field gel electrophoresis. †Calculated as 1 – *D* = (Σ*n*(*n –* 1))/(*N*(*N –* 1)), where n is number of isolates of each subtype and *N* is total number of isolates of a serovar. A value of 1 indicates infinite diversity, and a value of 0 indicates no diversity.

### Cluster and Outbreak Characteristics

During 2001–2007, a total of 376 *Salmonella* PFGE clusters were detected; they represented 1,399 (35%) isolates. Thirty-two (8.5%) clusters were excluded from analysis (21 secondary clusters, 7 clusters in which a hotline call directly contributed to identification of an outbreak, and 4 probable outbreak clusters). Forty-three (12.5%) of the 344 clusters included in the analysis were solved.

During 2001–2007, a total of 65 confirmed *Salmonella* outbreaks involving Minnesota cases were identified; these represented 502 (12.5%) isolates. Twenty-two (34%) outbreaks were excluded from analysis (6 were multistate outbreaks in which only 1 case was identified in Minnesota; in 7 outbreaks, a hotline call contributed to identification of the outbreak; 1 was an outbreak was not detected by PFGE; 4 were outbreaks that did not have cases that met the cluster definition; and 4 outbreaks were considered probable). The remaining 43 outbreaks, representing 287 (7%) isolates, were included in the analysis and were composed of 35 foodborne, 6 person-to-person, and 2 animal contact outbreaks. Of these 43 outbreaks, 30 (70%) involved 1 facility (restaurant, daycare center, school) or event and therefore were classified as point source. Thirteen (30%) involved commercially distributed food items at multiple points of sale (grocery stores, restaurants) and therefore were classified as non–point source. The median cluster size of point source outbreaks was 3 cases, and the median cluster size of non-point source outbreaks was 5 cases (p<0.01, by Wilcoxon rank-sum test). The median cluster density was 6 days for point source and non-point source outbreaks (p = 0.74 by Wilcoxon rank-sum test).

### Temporal Trends

**Figure 2:**
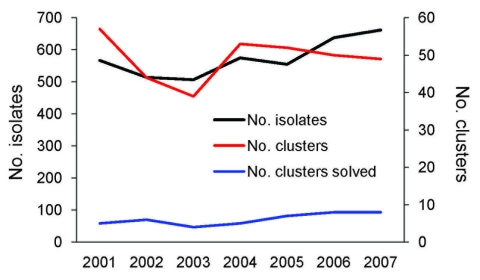
Temporal trends in number of *Salmonella enterica* isolates, number of clusters, and number of clusters solved (i.e., result in identification of a confirmed outbreak), Minnesota, USA, 2001–2007.

During the study period, the median number of *Salmonella* isolates subtyped per year was 567 (range 507–662 isolates). The median number of *Salmonella* clusters per year was 50 (range 44–57 clusters). The median number of confirmed *Salmonella* outbreaks per year was 6 (range 4–8 outbreaks). There were no statistically significant trends in the proportion of *Salmonella* clusters that resulted in identification of a confirmed outbreak (p = 0.20) (Figure 2).

### Cluster Serovar and Cluster Serovar Diversity

Clusters of the common *Salmonella* serovars Newport, Heidelberg, and Montevideo had 2.7× higher odds of being solved than did clusters of the very common serovars Enteritidis and Typhimurium (Table 2). The proportion of uncommon serovar clusters that were solved did not differ significantly from the proportion of very common or common serovar clusters that were solved (Table 2). Low clonality serovar clusters were not significantly more likely to be solved than highly clonal serovar clusters (OR 1.6, 95% CI 0.8–3.1).

**Table 2:** Univariate association between *Salmonella enterica* serovar frequency, cluster size, cluster density, and cluster being solved, Minnesota, USA, 2001–2007*

Characteristic	No. (%) solved clusters	No. unsolved clusters	Odds ratio (95% confidence interval)
Serovar			
Very common†	22 (10)	203	Referent
Common‡	11 (23)	37	2.74 (1.23–6.13)
Uncommon§	10 (14)	61	1.51 (0.68–3.37)
Total	43 (13)	301	
Cluster size¶			
2	16 (8)	194	Referent
3	8 (15)	47	2.06 (0.83–5.11)
4	7 (24)	22	3.86# (1.43–10.40)
>5	12 (24)	38	3.83 (1.68–8.74)
Total	43 (13)	301	
Cluster density, d**			
0	5 (71)	2	25.8 (3.42–195.37)
1–7	16 (33)	33	5.01 (1.33–18.89)
8–14	11 (22)	40	2.84 (0.73–11.07)
>15	3 (9)	31	Referent
Total	35 (25)	106	

*A solved cluster is one that results in identification of a confirmed outbreak. †*S. enterica* serovars Typhimurium and Enteritidis. ‡*S. enterica* serovars Newport, Heidelberg, and Montevideo. §All other serovars. ¶Significant Mantel-Haenszel χ^2^ test result for trend (p<0.001). #Clusters of 4 cases compared with clusters of 3 cases odds ratio 1.87, 95% confidence interval 0.52–6.66. **Cluster density measured as the number of days from receipt of first cluster case to third case received at the Minnesota Department of Health Public Health Laboratory.

### Cluster Subtype

No significant associations between the subtype frequency of a cluster and a cluster being solved were observed. Uncommon serovar Enteritidis subtype clusters were not significantly more likely to be solved than were common subtype clusters (OR 1.4, 95% CI 0.4–5.1). Uncommon serovar Typhimurium subtype clusters were not significantly more likely to be solved than were common subtype clusters (OR 0.9, 95% CI 0.3–3.2).

### Cluster Size

The probability of a cluster being solved increased significantly as the number of cluster cases increased (Mantel-Haenszel χ^2^ for trend 13.7, p<0.001) (Table 2). The odds of solving a cluster of >5 cases were 3.8× higher than the odds of solving a cluster of 2 cases. Clusters of 4 cases were 3.9× more likely to be solved than were clusters of 2 cases. Twenty-four percent of clusters with >4 cases were solved (Table 2). Clusters of 3 cases were 2.1× more likely to be solved than clusters of 2 cases, but the difference was not statistically significant. There was statistical evidence of a nonlinear relationship between cluster size and solving the cluster (Wald χ^2^ for interaction 5.0, p = 0.03). The dose response between cluster size and solving a cluster plateaued after a cluster size of 4.

### Cluster Case Density

The proportion of clusters solved increased significantly as the density of cluster cases increased (Mantel-Haenszel χ^2^ for trend, 12.7, p<0.001) (Table 2). The odds of solving a cluster if the first 3 case isolates were received on the same day were 25.8× higher than the odds of solving a cluster in which the first 3 case isolates were received during a period >14 days (Table 2). The odds of solving a cluster if the first 3 case isolates were received within 1–7 days were 5.0× higher than the odds of solving a cluster in which the first 3 case isolates were received during a period >14 days. Clusters in which the first 3 case isolates were received within 8–14 days were 2.8× more likely to be solved than clusters in which the first 3 case isolates were received during a period >14 days, but the difference was not statistically significant (Table 2). There was statistical evidence of a nonlinear relationship between cluster case density and solving the cluster (Wald χ^2^ for interaction, 6.96, p<0.01).

### Cluster Investigation Threshold

During June–December 2008, 10 MDH staff interviewed 214 persons with *Salmonella* infections and recorded the time required to complete the MDH standard questionnaire. Interview times did not vary between interviewers. The median interview time was 27 minutes (range 13–56 minutes). Therefore, conducting standard interviews of all cases in the 344 clusters of >2 cases (n = 1,182 [31%] cases) required an estimated 76 interview hours/year. This threshold detected all 43 outbreaks identified through routine laboratory surveillance during the study period and resulted in a cluster investigation positive predictive value (percentage of clusters investigated that were solved) of 13% (Table 3). Other cluster investigation thresholds had outbreak detection sensitivities of 53%–81% and positive predictive values of 23%–28% (Table 3).

**Table 3:** Comparison of *Salmonella enterica* cluster investigation thresholds, Minnesota, USA, 2001–2007*

Cluster investigation threshold	No. isolates represented in clusters	All *Salmonella* isolates, % (n = 3,803†)	Estimated interview time, h/y‡	No. (%) outbreak clusters meeting threshold	Cluster investigation PPV
All clusters (n = 344)	1,182	31	76	43 (100)	13
Clusters >3 cases (n = 152)	778	20	50	35 (81)	23
Clusters >4 cases (n = 83)	601	16	39	23 (53)	28
Clusters with a density of 0–14 d§ (n = 119)	633	17	41	32 (74)	27
Clusters >4 cases or with a density of 0–7 d§ (n = 100)	652	17	42	28 (65)	28

*PPV, positive predictive value. †A total of 215 isolates associated with excluded clusters were removed from study isolate total (n = 4,018). ‡Based on a 27-min median interview time per case-patient. §Density defined as the number of days from receipt of first cluster case isolate to third case isolate received at the Minnesota Department of Health Public Health Laboratory.

## Discussion

During the study period, 344 *Salmonella* PFGE clusters were identified and 43 (13%) were solved. Cluster size and cluster case density were the most useful predictors of a cluster being solved. The proportion of clusters that were solved increased as the number of cases in the cluster increased (up to 4 cases). The association was not linear and the percentage solved did not increase further for clusters with >5 cases. The observed association is logical because as the number of cluster cases increases, the amount of epidemiologic data available for evaluation also increases. Our results suggest that public health officials should not wait to investigate *Salmonella* clusters if >4 cluster cases have been received.

The ability to solve a cluster of cases of *Salmonella* infection was also strongly associated with the density of the cluster cases. The proportion of clusters that were solved increased as the density of the cluster cases increased, but this relationship was not linear. This association is also logical. Dense clusters increase the likelihood that the cluster cases are epidemiologically linked rather than unrelated sporadic cases. In addition, dense clusters also likely signal larger outbreaks. Our results demonstrated a clear increase in the success of solving clusters in which the first 3 case isolates were received within 7 days.

In theory, PFGE subtyping is less useful for recognizing clusters of unusual serovars worth investigating. In the current study, clusters of the common serovars Newport, Montevideo, and Heidelberg were statistically more likely to be solved than clusters of the very common serovars Enteritidis and Typhimurium. However, clusters of uncommon serovars were not more likely to be solved than were clusters of common or very common serovars. It has been suggested that uncommon serovar clusters may be associated with uncommon food vehicles, which makes them more difficult to solve by using standard methods (*24*). The relationship between serovar frequency and the likelihood of solving a cluster is unclear and warrants further study.

The limited number of solved clusters prevented multivariate analysis from being used to characterize the independent effect of predictors and possible effect modification between predictors. However, comparing the magnitude of the estimated effect of cluster size and cluster case density suggests that cluster case density may be a more useful predictor of a cluster being solved.

The 22 confirmed outbreaks that were excluded from the analysis demonstrate the value for national collaboration such as PulseNet and use of outbreak detection methods in addition to PFGE clustering within a given state. Six outbreaks were solved in which Minnesota only had 1 case, which demonstrated the utility of molecular subtyping in detecting geographically dispersed outbreaks. For 7 confirmed outbreaks, a call placed to the MDH foodborne illness hotline contributed to identification of the outbreak and demonstrated the utility of complaint systems in detecting outbreaks.

Interviewing all persons with *Salmonella* infection required a median of 27 minutes per person with *Salmonella* infection when the MDH standard questionnaire was used. By extrapolation, MDH staff spent ≈244 hours/year conducting routine interviews of persons with *Salmonella* infections. This figure does not include time spent attempting to reach persons, gathering demographic information from clinicians, or reinterviewing persons for cluster investigations. We recommend interviewing all persons with *Salmonella* infection and investigating all PFGE clusters to identify as many outbreaks as possible. However, many health departments do not have the resources to interview all persons with *Salmonella* infection or investigate all small clusters. Rather, they must balance the time required for these efforts and the ability to detect outbreaks (*25*).

Incorporating a cluster investigation threshold on the basis of cluster size and cluster case density can decrease the number of unsuccessful cluster investigations and conserve public health resources. However, this approach would also reduce the number of outbreaks that would be identified. One reason for this finding is that outbreaks that are manifested as smaller, less dense clusters would not be investigated. Another potential disadvantage of a cluster threshold approach is that delay of interviews until a cluster is solved can decrease the quality of exposure information obtained and therefore the likelihood that the cluster will be solved (*12*).

Four confirmed outbreaks during the study did not meet the cluster definition, and many confirmed outbreaks had cases that were outside the cluster definition. This finding is an important reminder that lack of temporal clustering does not eliminate the possibility of an outbreak. Increasing the period covered by a cluster definition will yield the benefit of solving more outbreaks. However, more resources will be expended conducting unsuccessful cluster investigations. The results of this study suggest that the use of a 2-week cluster window is sufficiently sensitive to detect most outbreaks. However, in practice, MDH epidemiologists do not use a strict 2-week cluster window when investigating clusters. Instead, all persons with *Salmonella* infection are interviewed and cases with matching PFGE patterns are often compared even if the second case is received >2 weeks after the first case.

The potential utility of the cluster investigation thresholds reported is based on the characteristics of the population of Minnesota and MDH surveillance methods: conducting real-time PFGE subtyping of all *Salmonella* isolates, interviewing all case-patients in real time by using a detailed exposure questionnaire from a central location for the entire state, and investigating clusters by using an iterative model (*19**–**21*). These factors aid in the timeliness of outbreak detection and investigation in Minnesota. These results may not be applicable in jurisdictions in which PFGE is not conducted in real time or batching of PFGE isolates occurs. Additional studies at the national level and in other states are needed to understand surveillance characteristics in other states and determine useful predictors of multistate clusters being solved.

Although successful cluster investigations will depend on the experience and ability of public health staff involved, this study demonstrates the increased probability of a cluster being solved as the number of cases in a cluster increases and as the cluster density increases. Specifically, investigation of PFGE clusters of >4 *Salmonella* case isolates and clusters in which the first 3 cases were received at the MDH PHL within 1 week yielded a major benefit in terms of outbreak identification. These results establish a benchmark for surveillance of *Salmonella* infections, and may provide a basis for investigating clusters of *Salmonella* cases for public health agencies with limited resources.

## References

[1] Voetsch AC, Van Gilder TJ, Angulo FJ, Farley MM, Shallow S, Marcus R, FoodNet estimate of the burden of illness caused by nontyphoidal *Salmonella* infections in the United States. Clin Infect Dis. 2004;38(Suppl 3):S127–34. 10.1086/381578 15095181

[2] Mead PS, Sultsker L, Dietz V, McCaig LF, Bresee JS, Shapiro C, Food-related illness and death in the United States. Emerg Infect Dis. 1999;5:607–25. 10.3201/eid0505.990502 10511517 PMC2627714

[3] Salmonellosis. In: Heymann DL, Thuriaux MC, editors. Control of communicable diseases manual. 18th ed. Washington: United Book Press; 2004. p. 469–73.

[4] Olsen SJ, Bishop R, Brenner FW, Roels TH, Bean N, Tauxe RV, The changing epidemiology of salmonella: trends in serotypes isolated from humans in the United States, 1987–1997. J Infect Dis. 2001;183:753–61. 10.1086/318832 11181152

[5] *Salmonella* infections. In: Pickering LK, Baker CJ, Kimberlin DW, Long SS, editors. Red book: 2006 report of the Committee on Infectious Diseases. 27th ed. Elk Grove Village (IL): American Academy of Pediatrics, 2006. p. 584–89.

[6] Swaminathan B, Barrett TJ, Hunter SB, Tauxe RV. CDC PulseNet Task Force. PulseNet: the molecular subtyping network for foodborne bacterial disease surveillance, United States. Emerg Infect Dis. 2001;7:382–9. 11384513 10.3201/eid0703.010303 PMC2631779

[7] Swaminathan B, Barrett TJ, Fields P. Surveillance for human *Salmonella* infections in the United States. J AOAC Int. 2006;89:553–9. 16640306

[8] Tauxe RV. Molecular subtyping and the transformation of public health. Foodborne Pathog Dis. 2006;3:4–8. 10.1089/fpd.2006.3.4 16602974

[9] Allos BM, Moore MR, Griffin PM, Tauxe RV. Surveillance for sporadic foodborne disease in the 21st century: the FoodNet perspective. Clin Infect Dis. 2004;38(Suppl 3):S115–20. 10.1086/381577 15095179

[10] Barrett TJ, Gerner-Smidt P, Swaminathan B. Interpretation of pulsed-field gel electrophoresis patterns in foodborne disease investigations and surveillance. Foodborne Pathog Dis. 2006;3:20–31. 10.1089/fpd.2006.3.20 16602976

[11] Buehler JW, Hopkins RS, Overhage JM, Sosin DM, Tong V; CDC Working Group. Framework for evaluating public health surveillance systems for early detection of outbreaks: recommendations form the CDC Working Group. MMWR Recomm Rep. 2004;53(RR-5):1–11. 15129191

[12] Hedberg CW, Greenblatt JR, Matyas BT, Lemmings J, Sharp DJ, Skibicki RT, Timeliness of enteric disease surveillance in 6 US states. Emerg Infect Dis. 2008;14:311–3. 10.3201/eid1402.070666 18258128 PMC2600210

[13] Lynch M, Painter J, Woodruff R, Braden C. Surveillance for foodborne-disease outbreaks—United States, 1998–2002. MMWR Surveill Summ. 2006;55(SS10):1–42. 17093388

[14] Hedberg CW, Besser JM. Commentary: cluster evaluation, PulseNet, and public health pracitce. Foodborne Pathog Dis. 2006;3:32–5. 10.1089/fpd.2006.3.32 16602977

[15] Council to Improve Foodborne Outbreak Response (CIFOR). Guidelines for foodborne disease outbreak response. Atlanta: Council of State and Territorial Epidemiologists; 2009.

[16] Hedberg CW, Jacquet C, Goulet V. Surveillance of listeriosis in France, 2000–2004: evaluation of cluster investigation criteria. Presented at the 16th International Symposium on Problems of Listeriosis. Savannah (GA) USA; 2007 Mar 20–23 [cited 20101 Jul 8]. http://www.aphl.org/profdev/conferences/proceedings/Documents/2007_ISOPOL/Surveillance_of_Listeriosis_in_France.pdf

[17] Reportable disease rule. Minnesota Department of Health. 2009 Jun 23 [cited 2010 Jul 8]. http://www.health.state.mn.us/divs/idepc/dtopics/reportable/rule/rule.html

[18] Ribot EM, Fair MA, Gautom R, Cameron DN, Hunter SB, Swaminathan B, Standardization of pulsed-field gel electrophoresis protocols for the subtyping of *Escherichia coli* O157:H7, *Salmonella*, and *Shigella* for PulseNet. Foodborne Pathog Dis. 2006;3:59–67. 10.1089/fpd.2006.3.59 16602980

[19] Smith KE, Medus C, Meyer SD, Boxrud DJ, Leano F, Hedberg CW, Outbreaks of salmonellosis in Minnesota (1998 through 2006) associated with frozen, microwaveable, breaded, stuffed chicken products. J Food Prot. 2008;71:2153–60. 18939771 10.4315/0362-028x-71.10.2153

[20] Centers for Disease Control and Prevention. Multistate outbreak of *Salmonella* infections associated with frozen pot pies—United States, 2007. MMWR Morb Mortal Wkly Rep. 2008;57:1277–80. 19037195

[21] Hedican E, Hooker C, Jenkins T, Medus C, Jawahir S, Leano F, Restaurant *Salmonella* Enteritidis outbreak associated with an asymptomatic infected food worker. J Food Prot. 2009;72:2332–6. 19903397 10.4315/0362-028x-72.11.2332

[22] Bender JB, Hedberg CW, Boxrud DJ, Besser JM, Wicklund JH, Smith KE, Use of molecular subtyping in surveillance for *Salmonella enterica* serotype Typhimurium. N Engl J Med. 2001;344:189–95. 10.1056/NEJM200101183440305 11172141

[23] Boxrud D, Pederson-Gulrud K, Wotton J, Medus C, Lyszkowicz E, Besser J, Comparison of multiple-locus variable-number tandem repeat analysis, pulsed-field gel electrophoresis, and phage typing for subtype analysis of *Salmonella enterica* serotype Enteritidis. J Clin Microbiol. 2007;45:536–43. 10.1128/JCM.01595-06 17151203 PMC1829081

[24] Lynch MF, Tauxe RV, Hedberg CW. The growing burden of foodborne outbreaks due to contaminated fresh produce: risks and opportunities. Epidemiol Infect. 2009;137:307–15. 10.1017/S0950268808001969 19200406

[25] Hoffman RE, Greenblatt J, Matyas BT, Sharp DJ, Esteban E, Hodge K, Capacity of state and territorial health agencies to prevent foodborne illness. Emerg Infect Dis. 2005;11:11–6. 15705316 10.3201/eid1101.040334 PMC3294323

